# Glycine decarboxylase is a target for transcriptional repressor Snail

**DOI:** 10.1186/2049-3002-2-S1-P83

**Published:** 2014-05-28

**Authors:** Guohua Chen, Stephanie Lucas, Jian Wang

**Affiliations:** 1 Department of Pathology, Wayne State University, Detroit, MI 48201, USA; 2 Department of Oncology, Detroit, MI 48201, USA

## Background

Aberrant glycine metabolism, an emerging hallmark of cancer, contributes to the aggressive proliferation [1] and invasion [2] of tumor cells. Glycine decarboxylase (GLDC) is the rate-limiting enzyme of glycine cleavage system that catabolizes glycine to feed one-carbon metabolism in mitochondria. A recent study suggests it plays a crucial role in tumorigenesis by promoting proliferation and pyrimidine synthesis of tumor initiation cells [3]. However, while its tumorigenic role is being unveiled, how it is de-regulated in tumor cells remains obscure. Here we present results showing that GLDC is a direct target for Snail, the key transcriptional repressor controlling the epithelial-mesenchymal transition (EMT) program during embryogenesis and tumor progression.

## Materials and method

We have generated clones of lung cancer A549 cells expressing Snail under the control of doxycycline-inducible promoter. Genes that are significantly down regulated in Snail-expressing cells are identified by comparative gene expression profiling with Illumina RNA-seq.

## Results

DAVID Gene Ontology analysis of Snail-responsive genes shows highest enrichment for cell adhesion, extracellular matrix, cytoskeleton, cell migration and proliferation, which validates the EMT functionality of transfected Snail. Interestingly, GLDC is found among the genes whose expressions are most down regulated in Snail-expressing cells. We further show that GLDC expression is significantly repressed at both mRNA and protein levels in response to Snail induction or by TGF-β treatment in a dose- and time-dependent manner in multiple cell lines. Sequence analysis reveals a couple of evolutionally conserved putative Snail-binding elements existing at the upstream proximal to the transcription start site within GLDC promoter. Using luciferase reporter assay and ChIP analysis, we show that wide-type not mutant Snail represses GLDC promoter activity by binding to GLDC promoter. Thus our genome-wide transcriptome screening and molecular characterization reveal GDLC as a new direct target for Snail. Given the critical role of Snail in endowing migratory and invasive cellular phenotypes, these results suggest the potential contribution of GLDC-mediated cellular metabolism to metastatic tumor progression.
